# Structural stability of DNA origami nanostructures under application-specific conditions

**DOI:** 10.1016/j.csbj.2018.09.002

**Published:** 2018-09-18

**Authors:** Saminathan Ramakrishnan, Heini Ijäs, Veikko Linko, Adrian Keller

**Affiliations:** a Technical and Macromolecular Chemistry, Paderborn University, Warburger Str. 100, 33098 Paderborn, Germany; b Biohybrid Materials, Department of Bioproducts and Biosystems, Aalto University, P. O. Box 16100, FI-00076 Aalto, Finland; c University of Jyväskylä, Department of Biological and Environmental Science, P. O. Box 35, FI-40014 Jyväskylä, Finland

**Keywords:** DNA origami, Stability, Denaturation, Drug delivery, Biophysics, Materials science

## Abstract

With the introduction of the DNA origami technique, it became possible to rapidly synthesize almost arbitrarily shaped molecular nanostructures at nearly stoichiometric yields. The technique furthermore provides absolute addressability in the sub-nm range, rendering DNA origami nanostructures highly attractive substrates for the controlled arrangement of functional species such as proteins, dyes, and nanoparticles. Consequently, DNAorigami nanostructures have found applications in numerous areas of fundamental and applied research, ranging from drug delivery to biosensing to plasmonics to inorganic materials synthesis. Since many of those applications rely on structurally intact, well-definedDNA origami shapes, the issue of DNA origami stability under numerous application-relevant environmental conditions has received increasing interest in the past few years. In this mini-review we discuss the structural stability, denaturation, and degradation of DNA origami nanostructures under different conditions relevant to the fields of biophysics and biochemistry, biomedicine, and materials science, and the methods to improve their stability for desired applications.

## Graphical abstract

**Figure:**
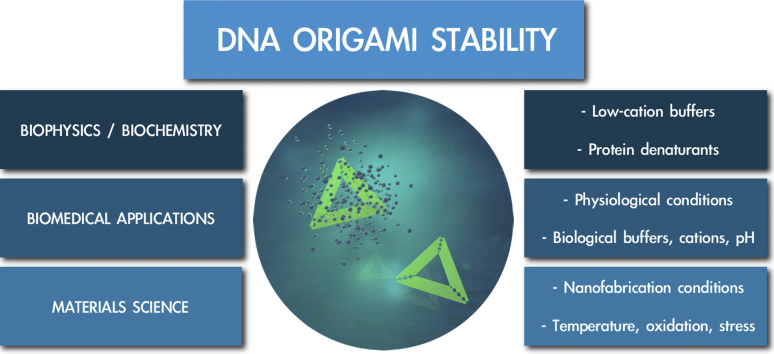

## 1 Introduction

During the brief history of structural DNA nanotechnology, the field has taken significant leaps from the very first branched DNA structures comprised of just a few DNA strands [1] to complex DNA shapes that are made of hundreds of DNA molecules [[2], [3], [4]]. As a result of this evolution we have acquired an ever-expanding toolbox of design techniques and software for creating custom and extremely precise nanostructures using DNA molecules as construction material. Even so, the ultimate goal in DNA nanotechnology is not only building these intricate DNA nanoshapes, but rather setting them in action. Very recently, the research field has reached the enabled state [5] at which biophysical, nanomedical, and materials science applications are increasingly coming into view [2,4,6].

A key player in the recently witnessed rapid development is the DNA origami technique [7], which is based on folding a long single-stranded scaffold strand into a desired shape with the help of dozens of short oligonucleotides. This technique provides a straightforward means to assemble user-definedDNA nanoshapes with sub-nanometer addressability [8]. Since its invention in 2006, the method has been further advanced and nowadays enables the fabrication of both 2D [7] and 3D structures [9,10] as well as curved and twisted shapes [11,12]. These relatively complex structures usually have molecular weights of a few megadalton and dimensions in the sub-100 nm range. Recent advances have extended this range to micrometer and gigadalton scales, respectively, using hierarchical assembly [13,14]. Other studies have introduced automated design strategies for wireframe-basedDNA origami [[15], [16], [17]] and demonstrated the mass production of DNA origami at affordable cost [18]. These recent advances will undoubtedly pave the way for many real-life applications, including biosensing [19,20], templated material synthesis [[21], [22], [23]], drug delivery [24,25], nanophotonics and plasmonics [26,27], nanoelectronics [[28], [29], [30]], and nanorobotics [[31], [32], [33]].

Although the abovementioned proof-of-concept implementations show the enormous potential of DNA origami, their utilization in the real-life applications requires detailed understanding of the structural and functional effects exerted by the surrounding environment. In particular, this is a pertinent issue for drug delivery, as the intended purpose of a DNA origami vehicle might easily get compromised in biological media. On the other hand, in materials science–related implementations, the DNA origami nanostructure itself may require specific conditions to reach its actual utility. Therefore, the structural stability of DNA origami is arguably a key issue that needs to be addressed to qualify DNA origami for numerous practical applications. In this mini-review, we discuss the DNA origami stability and degradation, as well as techniques to improve their resiliency, under a wide range of specific conditions related to a cornucopia of intriguing applications in biophysics and biochemistry (Section 2), biomedicine (Section 3), and materials science (Section 4).

## 2 Biophysical and biochemical applications

One of the first applications of DNA origami nanostructures was as molecular breadboards for the immobilization of chemical species and the visualization of chemical reactions at a single-molecule level using atomic force microscopy (AFM) [34]. Since then, numerous studies have employed DNA origami substrates in single-molecule studies of biophysical and biochemical processes [35,36], ranging from conformational transitions in DNA [[37], [38], [39], [40], [41]] to the movement of molecular motors [[42], [43], [44], [45]] to DNA radiation damage [[46], [47], [48], [49]]. Maintaining the structural integrity of the DNA origami may, however, pose significant limitations regarding their applicability in biophysical and biochemical studies. Most protocols for DNA origami assembly are based on Tris-acetate-EDTA (TAE) buffer supplemented with mM concentrations of Mg^2+^. These conditions, however, may not be compatible with the systems under investigation and interfere for instance with enzyme activity [50] or fluorescence emission [51]. On the other hand, deviations from these buffering conditions may compromise DNA origami integrity. Therefore, various studies have investigated the stability and denaturation of DNA origami nanostructures in different media.

**Fig. 1:**
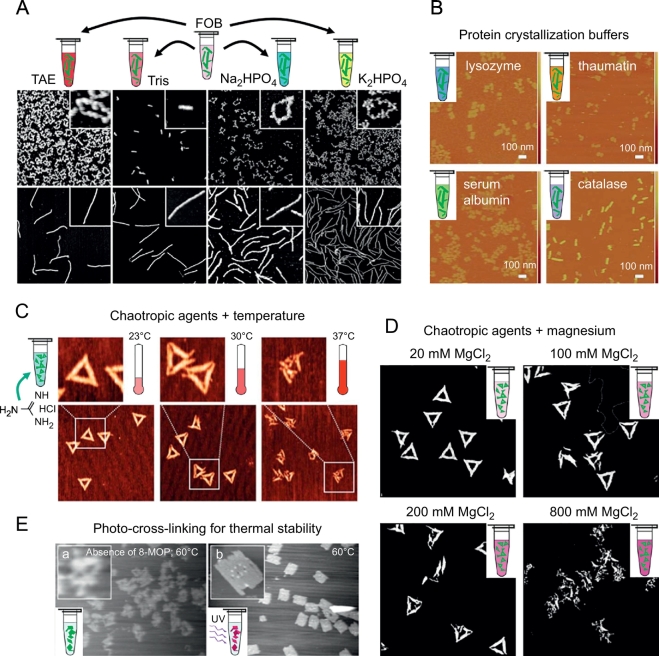
DNA origami under different biophysical and biochemical conditions. (A)24HBs (top panel) and 6HBs (bottom panel) in different low-magnesium buffers (FOB denotes folding buffer) [52]. (B)Tubular DNA origami structures in protein crystallization buffers of lysozyme, thaumatin, serum albumin and catalase [57]. (C)DNA origami triangles incubated at different temperatures in the presence of the chaotropic agent GdmCl at 6M concentration [61]. (D)DNA origami triangles in 4M GdmCl with varying MgCl_2_ concentrations [62]. (E)DNA origami sheets without (left) and with (right) 8-MOP-based photo-cross-linking at 60 °C [63]. (A)is reproduced with permission from Ref. [52]; copyright John Wiley & Sons 2018. (B)is reproduced with permission from Ref. [57]; published by Royal Society of Chemistry 2015. (C)is reproduced with permission from Ref. [61]; published by Royal Society of Chemistry 2016. (D)is reproduced with permission from Ref. [62]; copyright John Wiley & Sons 2017. (E)is reproduced with permission from Ref. [63]; copyright American Chemical Society 2011.

The need for Mg^2+^ concentrations in the mM range was for a long time considered a serious limitation for the application of DNA origami nanostructures, since cationic strength is fundamental to protect the DNA nanostructure from destabilization by electrostatic repulsion. However, such high Mg^2+^ concentrations are not a general requirement for ensuring DNA origami stability. It was demonstrated that DNA origami assembled in high Mg^2+^ concentrations can be transferred post-assembly into buffers containing Mg^2+^ concentrations in the low-μM range while maintaining their structural integrity, simply by spin-filtering [52]. Under these conditions, however, DNA origami stability is determined by the buffer composition. In particular, EDTA may complex and displace phosphate-bound Mg^2+^ ions and thereby denature the DNA origami. Phosphate ions on the other hand may interact with the bound Mg^2+^ ions and thus reduce their potential to screen electrostatic repulsion within the DNA origami construct. Most remarkably, these effects appear to be highly dependent on DNA origami shape and superstructure. For instance, Kielar et al. found 6-helix bundles absolutely stable in all buffers tested, while 24-helix bundles remained intact only in 10mM Tris (see Fig. 1A) [52]. Such superstructure-dependent effects on DNA origami stability are usually difficult to predict, and can arise from the contribution of multiple parameters, such as the chosen lattice type, compactness and charge density, structural flexibility, and strain associated with curvature [[52], [53], [54], [55], [56]].

Wang et al. studied the stability of DNA origami nanotubes in different buffers typically employed in the crystallization of various proteins, *i.e.*, lysozyme, thaumatin, human serum albumin, and catalase, and assessed the effects of cations, pH, protein precipitants, and type of the buffering agent [57]. While DNA origami assembly in these buffers did not result in any intact nanotubes, once-assembledDNA origami could successfully be transferred into catalase protein crystallization buffer (see Fig. 1B). A more detailed analysis revealed that the DNA origami nanotubes were denatured in Mg^2+^-free buffers containing 200mM Ca^2+^, K^+^, and NH_4_^+^, while they survived 200mM Na^+^. Additionally, the DNA origami nanotubes were found to be stable in Tris, HEPES, PEPES, and MES buffers, and in the presence of precipitating agents such as various alcohols and polymers, as well as NaCl at concentrations up to 3 M. They were also stable at pH values between 5 and 10, whereas DNA origami denaturation was observed at pH 4. Another study reported intact DNA origami nanostructures in the pH range from 4.5 to 9.5, which enabled their reversible multimerization by employing pH-sensitivei-motif and triplex DNA structures [58].

The study of protein folding and unfolding is an important research area in biophysics and increasingly investigated in single-molecule experiments using fluorescence techniques [59]. While such single-molecule measurements could benefit tremendously from using DNA origami nanostructures as substrates [41], they typically require the addition of high concentrations of denaturants such as urea or guanidinium chloride (GdmCl) to induce protein unfolding [60]. Maintaining DNA origami integrity under such denaturing conditions therefore is an important issue. As was demonstrated by Ramakrishnan et al., 2D DNA origami triangles remain mostly intact at room temperature in both 6 M urea and 6 M GdmCl for at least 24 h [61]. At elevated temperatures, however, DNA origami denaturation was observed and governed by the distribution of melting temperatures of the individual staple strands which may result in the structural collapse of the DNA origami at temperatures well below its global melting temperature (see Fig. 1C). Interestingly, DNAorigami stability at elevated temperatures can be improved in urea by increasing the concentration of cations in the buffer [62]. On the contrary, in GdmCl the elevated cation concentrations resulted in enhanced denaturation (see Fig. 1D), which was attributed to a salting-out of Gdm^+^ ions to the hydrophobic base stack of the DNA origami [62]. These effects are expected to again show a superstructure-dependence, which, however, may turn out even more complex due to the interplay of stabilizing and destabilizing contributions involving both inter- and intramolecular electrostatic interactions.

Finally, it has been demonstrated that DNA origami nanostructures can be stabilized by covalent cross-linking [63,64]. This was achieved by Rajendran et al. by exposing of DNA origami nanostructures to 8-methoxypsoralen (8-MOP) and subsequent irradiation with UVA light, which induced the formation of covalent 8-MOP-pyrimidineadducts [63]. This MOP-8-mediatedphoto-cross-linking resulted in a drastic increase in the melting temperature of the DNA origami by 30°C (see Fig. 1E). Gerling et al. utilized the UVB-induced formation of cyclobutane pyrimidine dimers (CPDs) between thymine-modifiedstaples within DNA origami nanostructures in order to increase their stability [64]. 3D DNA origami nanostructures cross-linked in this way were found to survive temperatures up to 90 °C. However, UVB and especially UVC irradiation was found to lead to DNA origami degradation at elevated irradiation doses, whereas long-wavelengthUVA irradiation did not result in significant DNA origami damage even at doses of 200 kJ/m^2^ [65].

## 3 Biomedical applications

Nanotechnology-based drug delivery systems [[66], [67], [68], [69]] have been widely studied to overcome limitations of conventional therapeutics, *e.g*., insolubility of drugs, specificity in targeting, multiple targeting, controlled release, and extended availability of the drug. However, there are also many disadvantages encountered in the various nanoparticle-based approaches, such as possible adverse immune effects and cytotoxicity, and experimental challenges in controlling the particle size and surface functionalization [70]. DNA origami nanostructures have the advantage that they are fully biocompatible, have defined sizes and shapes, and their surfaces can be modified in a precisely controlled manner. Moreover, DNA nanostructures can be loaded with drug molecules via different routes such as intercalation or DNA-conjugation. For example, doxorubicin-loadedDNA origami have been successfully used both *invitro* [71] and *invivo* [72].

When DNA origami are used in cellular environments, there are several factors that may affect their structural integrity. In cell cultures, tissue cultures, or *invivo*, DNA origami encounter low physiological cation concentrations and various different pH levels in distinct cellular compartments. In biological environments, DNA origami structures also face the active DNA-degrading machinery of cells. Digestion of genomic DNA is an important aspect in the homeostasis of living organisms [73,74] and takes place in the nucleus, cytoplasm, and extracellular space at various conditions involving different nucleases.

Castro et al. tested the stability of 18-, 24-, and 32-helix bundles against DNase I, T7 endonuclease I, T7 exonuclease, *Escherichia coli* exonuclease I, lambda exonuclease, and MseI restriction endonuclease [75]. The authors found that only DNase I and T7 endonuclease I degraded the test objects, while the other nucleases did not. This is a particularly important observation, since DNase I is the most abundant nuclease in blood and plasma [74,76]. In general, DNA origami have been reported to be more resistant to DNase I degradation than regular double-stranded DNA. The rate of digestion depends on the superstructure, so that closely-packed and compact structures are degraded more slowly [54,75]. Nevertheless, DNase I digestion still takes place in so short timescales that it represents one of the most crucial issues regarding the *invivo* stability and applicability of the structures.

Hahn et al. studied the effects of low magnesium concentration and nucleases on DNA origami stability in tissue culture media [53]. Out of the studied octahedra, 6-helix bundles, and 24-helix rods, only 6-helix bundles remained intact in low-magnesium tissue culture medium after 24 h incubation, despite its relatively high concentration of monovalent cations. Furthermore, all structures showed slow degradation in the presence of 10 % nuclease-containing fetal bovine serum (FBS), resulting in complete structural collapse after 24 h. Heat inactivation of the nucleases in FBS was found to reduce the rate of DNA origami degradation. Benson et al. and Veneziano et al. [15,17] reported that wireframe-basedDNA origami are stable in phosphate-buffered saline (PBS) and in Dulbecco’s Modified Eagle Media (DMEM) supplemented with FBS. Ahmadi et al., for one, showed that multilayer origami and wireframe structures were stable in DMEM with FBS [54]. In contrast, Jiang et al. reported that box-like 3D DNA origami nanostructures are extremely sensitive toward FBS and undergo almost instant degradation at serum concentrations as low as 0.1 % [77].

In drug delivery, the stability of DNA origami is also linked to their transfection efficiency and localization inside the cell. Structures that are not internalized may not be able to carry out their desired function and eventually, they will get degraded in the DNase I-rich extracellular environment. Even after a successful transfection, however, the DNA origami may be readily directed to lysosomal degradation pathways. Shen et al. thus investigated the stability and distribution of DNA origami nanotubes in breast cancer cells [78]. After 12 h of incubation, most of the DNA origami were localized in the lysosomes, where they remained intact up to 24 h. After 60 h, however, complete degradation of the DNA origami nanostructures was observed.

**Fig. 2:**
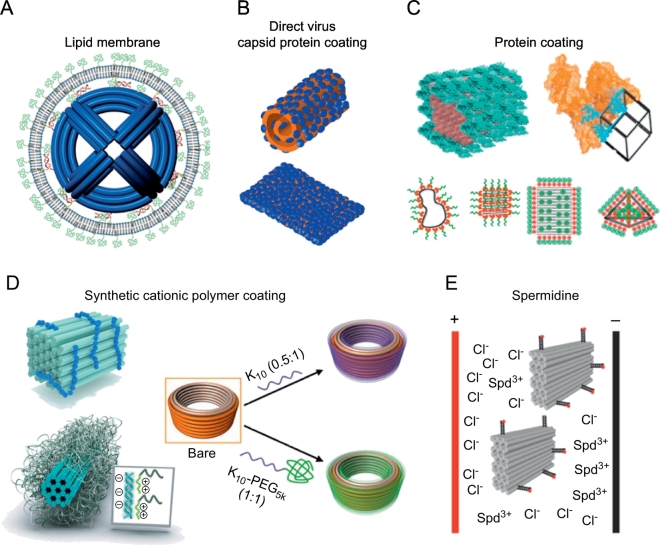
Coating and modifying DNA nanostructures for biomedical applications. (A) DNA origami with lipid bilayer coating [79]. (B) DNA origami encapsulated with CCMV capsid proteins [80]. (C) DNA origami coated with BSA-dendron conjugate (top left) [84]. DNA nanocage linked to BSA (top right) [81]. DNA strands, DNA origami sheets, and tetrahedron coated with peptide-polymer conjugates (low panel) [82,83]. (D) DNA origami shapes coated by synthetic cationic polymers; PEG-PDMAEMA-, PEG-oligolysine- and PEG-polylysine-based copolymers [85,87,88]. (E) Electrotransfection of DNA origami folded with Spd^3+^ [86]. (A) is reproduced with permission from Ref. [79]; published by American Chemical Society 2014. (B) is reproduced with permission from Ref. [80]; copyright American Chemical Society 2014. (C) (top left) is reproduced with permission from Ref. [84]; published by John Wiley & Sons 2017. (C) (top right) is reproduced with permission from Ref. [81]; copyright American Chemical Society 2017. (C) (bottom left) is reproduced with permission from Ref. [82]; copyright American Chemical Society 2017. (C) (bottom right) is reproduced with permission from Ref. [83]; copyright American Chemical Society 2017. (D) (top left) is reproduced with permission from Ref. [87]; published by Royal Society of Chemistry 2016. (D) (bottom left) is reproduced with permission from Ref. [85]; copyright John Wiley & Sons 2017. (D) (right) is reproduced with permission from Ref. [88]; published by Nature Publishing Group. (E) is reproduced with permission from Ref. [86]; copyright American Chemical Society 2016.

Being aware of the stability-related challenges associated with biological environments, many recent studies have focused on developing methods to increase the stability of DNA origami nanostructures in biological applications. Most notably, these approaches include various methods to coat the structures non-covalently with other molecules (Fig. 2). DNA origami have been encapsulated with virus-mimicking lipid bilayers (Fig. 2A) [79], virus capsid proteins (from chlorotic cowpea mottle virus (CCMV)) using electrostatic interactions (Fig. 2B) [80], other proteins, such as bovine serum albumin (BSA), protein-polymer, and protein-dendron conjugates (Fig. 2C) [[81], [82], [83], [84]], and variouscationic polymers such as polyethylene glycol (PEG) –oligo- and polylysines, chitosan, polyethylenimine (PEI) and PEG-poly(2-dimethylaminoethyl methacrylate (PEG-PDMAEMA)) –based copolymers (Fig. 2D) [54,[85], [86], [87], [88]]. Shielding provided by the coating agents has been demonstrated to result in restricted accessibility of enclosed cargo [87], increased DNA origami resistance against DNase I digestion [54,79,[83], [84], [85],^88^], stability in low-salt conditions [54,83,85,88], and attenuated immune response [79,84]. Transfection efficiency of DNA origami structures has been shown to depend on the mass and shape of the structures so that compact, low-aspect ratio structures are more efficiently internalized [89,90], but in general, highly polar DNA structures cross lipid bilayers weakly and have relatively low transfection efficiency. Several of these coating strategies have also been shown to improve transfection efficiency of the DNA structures [79,80,84]. In addition, DNA origami folded in spermidine (Spd^3+^) containing buffers have been shown to withstand high electric field pulses, so that these structures could be transported into both mammalian and bacterial cells using electrotransfection (Fig. 2E) [86].

## 4 Materials science applications

DNA origami nanostructures are also increasingly employed in various materials science applications. Many studies have used DNA origami nanostructures as templates or lithography masks in order to transfer their shapes into other biological [22,[91], [92], [93]], organic [[94], [95], [96], [97]], and especially inorganic materials [21,23,29,30,[98], [99], [100], [101]]. Even if this requires conditions that deviate from the usual solution conditions, the shapes of the DNA origami templates are often transferred in a single processing step, so that their structural stability is usually of little concern. However, some applications require more complex transfer procedures involving several rather harsh processing steps that may damage the DNA origami nanostructures. For instance, Jin et al. have transferred DNA nanostructure shapes into graphene by reactive ion etching [100]. Since ion etching destroyed the DNA templates, they had to be metallized prior to the etching step. DNA origami metallization, however, may not only alter the size and shape of the original structures, but also introduce undesired and sometimes harmful contaminations, for instance in semiconductor device fabrication. Fortunately, DNA origami nanostructures have been found to remain structurally intact under a large variety of conditions frequently encountered in lithographic and thin film processing.

**Fig. 3:**
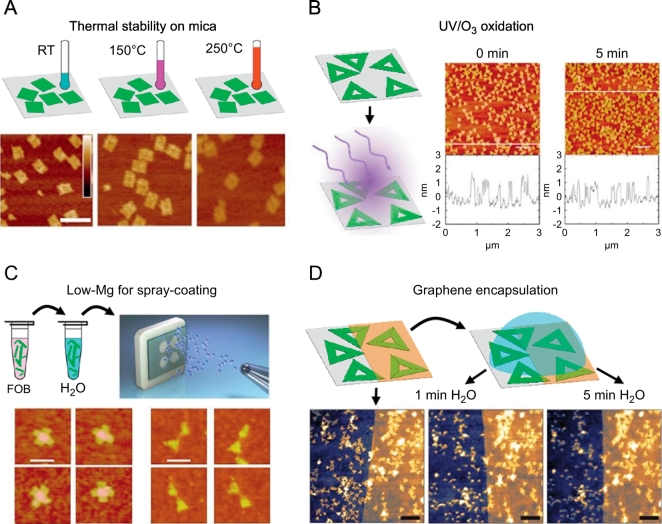
DNA origami under different conditions relevant for materials science applications. (A) Adsorbed DNA origami at room temperature (left), at 150 °C (middle) and 250 °C (right) [102]. (B) Adsorbed DNA origami triangles before (left) and after 5 min exposure to UV/ozone (right) [103]. (C) Cross and bowtie DNA origami shapes are transferred from folding buffer (FOB) to H_2_O (low-μM Mg^2+^ concentrations) and spray-deposited onto substrates [107]. (D) Graphene encapsulation protects immobilized triangular DNA origami from exposure to H_2_O [109]. (A) is reproduced with permission from Ref. [102]; copyright American Vacuum Society 2014. (B) is reproduced with permission from Ref. [103]; copyright American Chemical Society 2014. (C) is reproduced with permission from Ref. [107]; published by Nature Publishing Group 2015. (D) is reproduced with permission from Ref. [109]; published by IOP Publishing 2016.

Pillers et al. for instance investigated the thermal stability of DNA origami nanostructures adsorbed to mica surfaces [102]. They found that the adsorbed DNA origami could withstand temperatures of 150°C in air for at least 45 min. At higher temperatures of 250 °C, however, decomposition of the DNA origami was observed (see Fig. 3A). A broader study of the stability of DNA origami nanostructures adsorbed on SiO_2_ surfaces was carried out by Kim et al. who investigated the effects of several chemical environments relevant for various lithographic processing and film deposition techniques [103]. The authors found adsorbed DNA origami triangles to survive heating in air and argon atmospheres up to 200 °C without visual signs of decomposition. Prolonged exposure of the DNA origami triangles up to at least 24 h to organic solvents (hexane, ethanol, and toluene) did not result in any significant changes in DNA origami structure. Exposure to deionized water, on the other hand, did not only induce DNA origami desorption from the SiO_2_ surface but also sometimes led to significant damage. Similar observations were made for incubation in NaCl solutions but with additional salt accumulation on the DNA origami. Low pH values below 4 were found to denature the DNA origami triangles whereas their structural integrity was well preserved up to pH 11. Most remarkably, under highly oxidizing conditions, *i.e.* exposure to UV/ozone, the DNA origami nanostructures were found to maintain their original shape for several minutes (see Fig. 3B).

Immobilization of DNA origami on SiO_2_ surfaces is a prerequisite for many applications in materials science and typically achieved by addition of high concentrations (0.1–0.5 M)of Mg^2+^ ions [101,[104], [105], [106]]. This, however, may result in residual salt deposits on the surfaces, which can interfere with subsequent processing steps. In order to avoid salt residues, Linko et al. employed a spray coating technique to homogenously deposit DNA origami nanostructures at low densities over large surface areas [107]. To this end, the Mg^2+^-containing assembly buffer was exchanged against pure water right before the spray coating step, resulting in residual Mg^2+^ concentrations in the low-μM range. Despite being immersed in essentially salt-free medium and sprayed onto the substrate at 3bar pressure, the DNA origami nanostructures were found to remain structurally intact (see Fig. 3C).

Despite the extraordinarily high stability of adsorbed DNA origami nanostructures immobilized at a solid substrate discussed above, two key issues have been identified that limit post-immobilization processing of DNA origami: their limited mechanical stability and their sensitivity toward exposure to pure water which rapidly induces desorption and shape distortions. Both effects for instance limit the repeated application of DNA nanostructure masters in polymer imprinting [94]. This problem, however, may be overcome by coating the immobilized DNA origami nanostructures with a thin protective film. To this end, Kim et al. employed atomic layer deposition (ALD) of thin (2–5 nm) Al_2_O_3_ films and showed that such films not only perfectly reproduce the shape of DNA origami triangles and DNA nanotubes, but also protect the DNA nanostructure masters during repeated imprinting, washing with pure water, and extended UV/ozone exposure for 1 h [108]. In a similar approach, Matković et al. used single-layer graphene as a protective layer on top of DNA origami nanostructures adsorbed on SiO_2_ surfaces [109]. This graphene layer reproduced the triangular shape of the DNA origami remarkably well, while at the same time protecting them against mechanical damage during contact-mode atomic force microscopy imaging, as well as exposure to pure water for at least 30min (see Fig. 3D).

## 5 Summary and perspective

DNA origami nanostructures are nowadays employed in a multitude of applications in which they may encounter very different environments. Just as numerous and diverse are the effects that these environments may have on the structural and functional properties of a given DNA origami. In particular, DNA in general shows an intrinsic sensitivity toward temperature, cation concentration, and nuclease attack. Consequently, a number of protection strategies employing various coatings and chemical modifications have been developed in order to ensure structural stability of DNA origami under such highly relevant conditions.

Nevertheless, DNA origami have been found surprisingly stable under a number of rather extreme conditions. In particular, dry DNA origami adsorbed on a solid substrate have been shown to survive a number of remarkably harsh environmental conditions, including high temperatures and UV/ozone exposure [102,103], which qualifies them for numerous applications in templated materials synthesis. DNA origami may maintain their structural integrity also under highly denaturing conditions in solution, for instance in the presence of highly concentrated chaotropic agents [61]. However, the interaction of such chaotropic denaturants with DNA origami nanostructures appears to be highly complex and exhibits some rather unexpected features, such as denaturant-specific modes of attack [61] and the gradual destabilization of DNA origami in GdmCl by increasing cation concentrations [62]. Understanding these complex processes will require further experimental and theoretical investigations of the molecular mechanisms involved.

In stark contrast to the observations above, more natural/biological conditions may result in rapid DNA origami degradation, as exemplified by the almost instantaneous disintegration of a 3D DNA origami box in 0.1 % serum [77]. This is even more surprising when compared to the study by Hahn et al., who observed a much slower digestion of several 3D DNA origami in 10 % serum over several hours [53]. These observations suggest that DNA origami design and superstructure may play a dominant role in DNA origami digestion by modulating nuclease attack [110]. Although DNA origami superstructure has already been identified to govern nanostructural stability under low-magnesium conditions [52,53], the nature of these effects is so far only poorly understood. Elucidating the molecular mechanisms of such superstructure-dependent effects holds the promise of the rational design of nuclease-resistantDNA origami nanostructures [111] with a broad range of applications in biomedical science.

## Declarations of interest

None.

## Acknowledgements

This work was funded by the Academy of Finland (grant number 286845), Jane and Aatos Erkko Foundation, and Sigrid Jusélius Foundation. It was carried out under the Academy of Finland Centers of Excellence Programme (2014–2019). Financial support from Deutsche Forschungsgemeinschaft (to A.K., grant number KE 1944/2-1) is gratefully acknowledged.

## References

[1] Seeman N.C. (1982). Nucleic acid junctions and lattices. J Theor Biol.

[2] Seeman N.C., Sleiman H.F. (2017). DNA nanotechnology. Nat Rev Mater.

[3] Nummelin S., Kommeri J., Kostiainen M.A., Linko V. (2018). Evolution of structural DNA nanotechnology. Adv Mater.

[4] Bathe M., Rothemund P.W.K. (2017). DNA Nanotechnology: A foundation for Programmable Nanoscale Materials. MRS Bull.

[5] Linko V., Dietz H. (2013). The enabled state of DNA nanotechnology. Curr Opin Biotechnol.

[6] Hong F., Zhang F., Liu Y., Yan H. (2017). DNA origami: scaffolds for creating higher order structures. Chem Rev.

[7] Rothemund P.W.K. (2006). Folding DNA to create nanoscale shapes and patterns. Nature.

[8] Funke J.J., Dietz H. (2016). Placing molecules with Bohr radius resolution using DNA origami. Nat Nanotechnol.

[9] Andersen E.S., Dong M., Nielsen M.M., Jahn K., Subramani R., Mamdouh W. (2009). Self-assembly of a nanoscale DNA box with a controllable lid. Nature.

[10] Douglas S.M., Dietz H., Liedl T., Högberg B., Graf F., Shih W.M. (2009). Self-assembly of DNA into nanoscale three-dimensional shapes. Nature.

[11] Dietz H., Douglas S.M., Shih W.M. (2009). Folding DNA into twisted and curved nanoscale shapes. Science.

[12] Han D., Pal S., Nangreave J., Deng Z., Liu Y., Yan H. (2011). DNA origami with complex curvatures in three-dimensional space. Science.

[13] Tikhomirov G., Petersen P., Qian L. (2017). Fractal assembly of micrometre-scaleDNA origami arrays with arbitrary patterns. Nature.

[14] Wagenbauer K.F., Sigl C., Dietz H. (2017). Gigadalton-scaleshape-programmableDNA assemblies. Nature.

[15] Benson E., Mohammed A., Gardell J., Masich S., Czeizler E., Orponen P. (2015). DNA rendering of polyhedral meshes at the nanoscale. Nature.

[16] Linko V., Kostiainen M.A. (2016). Automated design of DNA origami. Nat Biotechnol.

[17] Veneziano R., Ratanalert S., Zhang K., Zhang F., Yan H., Chiu W. (2016). Designer nanoscale DNA assemblies programmed from the top down. Science.

[18] Praetorius F., Kick B., Behler K.L., Honemann M.N., Weuster-Botz D., Dietz H. (2017). Biotechnological mass production of DNA origami. Nature.

[19] Ochmann S.E., Vietz C., Trofymchuk K., Acuna G.P., Lalkens B., Tinnefeld P. (2017). Optical nanoantenna for single molecule-based detection of zika virus nucleic acids without molecular multiplication. Anal Chem.

[20] Daems D., Pfeifer W., Rutten I., Saccà B., Spasic D., Lammertyn J. (2018). Three-dimensionalDNA origami as programmable anchoring points for bioreceptors in fiber optic surface plasmon resonance biosensing. ACS Appl Mater Interfaces.

[21] Shen B., Linko V., Tapio K., Pikker S., Lemma T., Gopinath A. (2018). Plasmonic nanostructures through DNA-assisted lithography. Sci Adv.

[22] Ramakrishnan S., Subramaniam S., Stewart A.F., Grundmeier G., Keller A. (2016). Regular nanoscale protein patterns via directed adsorption through self-assembledDNA origami masks. ACS Appl Mater Interfaces.

[23] Liu X., Zhang F., Jing X., Pan M., Liu P., Li W. (2018). Complex silica composite nanomaterials templated with DNA origami. Nature.

[24] Surana S., Shenoy A.R., Krishnan Y. (2015). Designing DNA nanodevices for compatibility with the immune system of higher organisms. Nat Nanotechnol.

[25] Linko V., Ora A., Kostiainen M.A. (2015). DNA nanostructures as smart drug-delivery vehicles and molecular devices. Trends Biotechnol.

[26] Liu N., Liedl T. (2018). DNA-assembled advanced plasmonic architectures. Chem Rev.

[27] Kuzyk A., Jungmann R., Acuna G.P., Liu N. (2018). DNA origami route for nanophotonics. ACS Photonics.

[28] Shen B., Linko V., Dietz H., Toppari J.J. (2015). Dielectrophoretic trapping of multilayer DNA origami nanostructures and DNAorigami-induced local destruction of silicon dioxide. Electrophoresis.

[29] Teschome B., Facsko S., Schönherr T., Kerbusch J., Keller A., Erbe A. (2016). Temperature-dependent charge transport through individually contacted DNAorigami-based Au nanowires. Langmuir.

[30] Geng Y., Pearson A.C., Gates E.P., Uprety B., Davis R.C., Harb J.N. (2013). Electrically conductive gold- and copper-metallizedDNA origami nanostructures. Langmuir.

[31] Thubagere A.J., Li W., Johnson R.F., Chen Z., Doroudi S., Lee Y.L. (2017). A cargo-sortingDNA robot. Science.

[32] Douglas S.M., Bachelet I., Church G.M. (2012). A logic-gated nanorobot for targeted transport of molecular payloads. Science.

[33] Ijäs H., Nummelin S., Shen B., Kostiainen M.A., Linko V. (2018). Dynamic DNA origami devices: from strand-displacement reactions to external-stimuli responsive systems. Int J Mol Sci.

[34] Voigt N.V., Tørring T., Rotaru A., Jacobsen M.F., Ravnsbaek J.B., Subramani R. (2010). Single-molecule chemical reactions on DNA origami. Nat Nanotechnol.

[35] Rajendran A., Endo M., Sugiyama H. (2012). Single-molecule analysis using DNA origami. Angew Chem Int Ed Engl.

[36] Bald I., Keller A. (2014). Molecular processes studied at a single-molecule level using DNA origami nanostructures and atomic force microscopy. Molecules.

[37] Rajendran A., Endo M., Hidaka K., Sugiyama H. (2014). Direct and single-molecule visualization of the solution-state structures of G-hairpin and G-triplex intermediates. Angew Chem Int Ed Engl.

[38] Sannohe Y., Endo M., Katsuda Y., Hidaka K., Sugiyama H. (2010). Visualization of dynamic conformational switching of the G-quadruplex in a DNA nanostructure. J Am Chem Soc.

[39] Tsukanov R., Tomov T.E., Berger Y., Liber M., Nir E. (2013). Conformational dynamics of DNA hairpins at millisecond resolution obtained from analysis of single-moleculeFRET histograms. J Phys Chem B.

[40] Tsukanov R., Tomov T.E., Masoud R., Drory H., Plavner N., Liber M. (2013). Detailed study of DNA hairpin dynamics using single-molecule fluorescence assisted by DNA origami. J Phys Chem B.

[41] Gietl A., Holzmeister P., Grohmann D., Tinnefeld P. (2012). DNA origami as biocompatible surface to match single-molecule and ensemble experiments. Nucleic Acids Res.

[42] Tomov T.E., Tsukanov R., Glick Y., Berger Y., Liber M., Avrahami D. (2017). DNA bipedal motor achieves a large number of steps due to operation using microfluidics-based interface. ACS Nano.

[43] Wickham S.F.J., Endo M., Katsuda Y., Hidaka K., Bath J., Sugiyama H. (2011). Direct observation of stepwise movement of a synthetic molecular transporter. Nat Nanotechnol.

[44] Yang Y., Goetzfried M.A., Hidaka K., You M., Tan W., Sugiyama H. (2015). Direct visualization of walking motions of photocontrolled nanomachine on the DNA nanostructure. Nano Lett.

[45] Tomov T.E., Tsukanov R., Liber M., Masoud R., Plavner N., Nir E. (2013). Rational design of DNA motors: fuel optimization through single-molecule fluorescence. J Am Chem Soc.

[46] Keller A., Bald I., Rotaru A., Cauët E., Gothelf K.V., Besenbacher F. (2012). Probing electron-induced bond cleavage at the single-molecule level using DNA origami templates. ACS Nano.

[47] Keller A., Rackwitz J., Cauët E., Liévin J., Körzdörfer T., Rotaru A. (2014). Sequence dependence of electron-inducedDNA strand breakage revealed by DNA nanoarrays. Sci Rep.

[48] Rackwitz J., Bald I. (2018). Low-energyelectron-induced strand breaks in telomere-derivedDNAsequences-influence of DNA sequence and topology. Chem Eur J.

[49] Rackwitz J., Kopyra J., Dąbkowska I., Ebel K., Ranković M.L., Milosavljević A.R. (2016). Sensitizing DNA towards low-energy electrons with 2-fluoroadenine. Angew Chem Int Ed Engl.

[50] Hallcher L.M., Sherman W.R. (1980). The effects of lithium ion and other agents on the activity of myo-inositol-1-phosphatase from bovine brain. J Biol Chem.

[51] Kring J.P., Williams J.N. (1955). Interference in the fluorometric analysis of pyridine nucleotides by certain ions. J Biol Chem.

[52] Kielar C., Xin Y., Shen B., Kostiainen M.A., Grundmeier G., Linko V. (2018). On the stability of DNA origami nanostructures in low-magnesium buffers. Angew Chem Int Ed Engl.

[53] Hahn J., Wickham S.F.J., Shih W.M., Perrault S.D. (2014). Addressing the instability of DNA nanostructures in tissue culture. ACS Nano.

[54] Ahmadi Y., de Llano E., Barišić I. (2018). (Poly)cation-induced protection of conventional and wireframe DNA origami nanostructures. Nanoscale.

[55] Kollmann F., Ramakrishnan S., Shen B., Grundmeier G., Kostiainen M.A., Linko V. (2018). Superstructure-dependent loading of DNA origami nanostructures with a groove-binding drug. ACS Omega.

[56] Opherden L., Oertel J., Barkleit A., Fahmy K., Keller A. (2014). Paramagnetic decoration of DNA origami nanostructures by Eu^3+^ coordination. Langmuir.

[57] Wang D., Da Z., Zhang B., Isbell M.A., Dong Y., Zhou X. (2015). Stability study of tubular DNA origami in the presence of protein crystallisation buffer. RSC Adv.

[58] Wu N., Willner I. (2016). pH-stimulated reconfiguration and structural isomerization of origami dimer and trimer systems. Nano Lett.

[59] Schuler B., Hofmann H. (2013). Single-molecule spectroscopy of protein folding dynamics--expanding scope and timescales. Curr Opin Struct Biol.

[60] Lo Nostro P., Ninham B.W. (2012). Hofmeister phenomena: An update on ion specificity in biology. Chem Rev.

[61] Ramakrishnan S., Krainer G., Grundmeier G., Schlierf M., Keller A. (2016). Structural stability of DNA origami nanostructures in the presence of chaotropic agents. Nanoscale.

[62] Ramakrishnan S., Krainer G., Grundmeier G., Schlierf M., Keller A. (2017). Cation-Induced stabilization and denaturation of DNA origami nanostructures in urea and guanidinium chloride. Small.

[63] Rajendran A., Endo M., Katsuda Y., Hidaka K., Sugiyama H. (2011). Photo-cross-linking-assisted thermal stability of DNA origami structures and its application for higher-temperatureself-assembly. J Am Chem Soc.

[64] Gerling T., Kube M., Kick B., Dietz H. (2018). Sequence-programmable covalent bonding of designed DNA assemblies. Sci Adv.

[65] Chen H., Li R., Li S., Andréasson J., Choi J.H. (2017). Conformational effects of UV light on DNA origami. J Am Chem Soc.

[66] Suri S.S., Fenniri H., Singh B. (2007). Nanotechnology-based drug delivery systems. J Occup Med Toxicol.

[67] Bayda S., Hadla M., Palazzolo S., Corona G., Toffoli G., Rizzolio F. (2018). Inorganic nanoparticles for cancer therapy: A transition from lab to clinic. Curr Med Chem.

[68] Palazzolo S., Bayda S., Hadla M., Caligiuri I., Corona G., Toffoli G. (2017). The clinical translation of organic nanomaterials for cancer therapy: a focus on polymeric nanoparticles, micelles, liposomes and exosomes. Curr. Med. Chem..

[69] Wang R., Billone P.S., Mullett W.M. (2013). Nanomedicine in action: an overview of cancer nanomedicine on the market and in clinical trials. J Nanomater.

[70] de Jong W.H., Borm P.J.A. (2008). Drug delivery and nanoparticles: Applications and hazards. Int J Nanomedicine.

[71] Jiang Q., Song C., Nangreave J., Liu X., Lin L., Qiu D. (2012). DNA origami as a carrier for circumvention of drug resistance. J Am Chem Soc.

[72] Zhang Q., Jiang Q., Li N., Dai L., Liu Q., Song L. (2014). DNA origami as an invivo drug delivery vehicle for cancer therapy. ACS Nano.

[73] Zhang J., Xu M. (2002). Apoptotic DNA fragmentation and tissue homeostasis. Trends Cell Biol.

[74] Samejima K., Earnshaw W.C. (2005). Trashing the genome: The role of nucleases during apoptosis. Nat Rev Mol Cell Biol.

[75] Castro C.E., Kilchherr F., Kim D.-N., Shiao E.L., Wauer T., Wortmann P. (2011). A primer to scaffolded DNA origami. Nat Methods.

[76] Kishi K., Yasuda T., Ikehara Y., Sawazaki K., Sato W., Iida R. (1990). Human serum deoxyribonuclease I (DNase I)polymorphism: pattern similarities among isozymes from serum, urine, kidney, liver, and pancreas. Am J Hum Genet.

[77] Jiang Z., Zhang S., Yang C., Kjems J., Huang Y., Besenbacher F. (2015). Serum-induced degradation of 3D DNA box origami observed with high-speed atomic force microscopy. Nano Res.

[78] Shen X., Jiang Q., Wang J., Dai L., Zou G., Wang Z.-G. (2012). Visualization of the intracellular location and stability of DNA origami with a label-free fluorescent probe. Chem Commun.

[79] Perrault S.D., Shih W.M. (2014). Virus-inspired membrane encapsulation of DNA nanostructures to achieve invivo stability. ACS Nano.

[80] Mikkilä J., Eskelinen A.-P., Niemelä E.H., Linko V., Frilander M.J., Törmä P. (2014). Virus-encapsulatedDNA origami nanostructures for cellular delivery. Nano Lett.

[81] Lacroix A., Edwardson T.G.W., Hancock M.A., Dore M.D., Sleiman H.F. (2017). Development of DNA nanostructures for high-affinity binding to human serum albumin. J Am Chem Soc.

[82] Hernandez-Garcia A., Estrich N.A., Werten M.W.T., van der Maarel J.R.C., LaBean T.H., de Wolf F.A. (2017). Precise coating of a wide range of DNA templates by a protein polymer with a DNA binding domain. ACS Nano.

[83] Estrich N.A., Hernandez-Garcia A., de Vries R., LaBean T.H. (2017). Engineered diblock polypeptides improve DNA and gold solubility during molecular assembly. ACS Nano.

[84] Auvinen H., Zhang H., Nonappa, Kopilow A., Niemelä E.H., Nummelin S. (2017). Protein coating of DNA nanostructures for enhanced stability and immunocompatibility. Adv. Healthc. Mater..

[85] Agarwal N.P., Matthies M., Gür F.N., Osada K., Schmidt T.L. (2017). Block copolymer micellization as a protection strategy for DNA origami. Angew Chem Int Ed Engl.

[86] Chopra A., Krishnan S., Simmel F.C. (2016). Electrotransfection of polyamine folded DNA origami structures. Nano Lett.

[87] Kiviaho J.K., Linko V., Ora A., Tiainen T., Järvihaavisto E., Mikkilä J. (2016). Cationic polymers for DNA origami coating - examining their binding efficiency and tuning the enzymatic reaction rates. Nanoscale.

[88] Ponnuswamy N., Bastings M.M.C., Nathwani B., Ryu J.H., Chou L.Y.T., Vinther M. (2017). Oligolysine-based coating protects DNA nanostructures from low-salt denaturation and nuclease degradation. Nat Commun.

[89] Schüller V.J., Heidegger S., Sandholzer N., Nickels P.C., Suhartha N.A., Endres S. (2011). Cellular immunostimulation by CpG-sequence-coatedDNA origami structures. ACS Nano.

[90] Bastings M.M.C., Anastassacos F.M., Ponnuswamy N., Leifer F.G., Cuneo G., Lin C. (2018). Modulation of the cellular uptake of DNA origami through control over mass and shape. Nano Lett.

[91] Aslan H., Krissanaprasit A., Besenbacher F., Gothelf K.V., Dong M. (2016). Protein patterning by a DNA origami framework. Nanoscale.

[92] Busuttil K., Rotaru A., Dong M., Besenbacher F., Gothelf K.V. (2013). Transfer of a protein pattern from self-assembledDNA origami to a functionalized substrate. Chem Commun.

[93] Sajfutdinow M., Uhlig K., Prager A., Schneider C., Abel B., Smith D.M. (2017). Nanoscale patterning of self-assembled monolayer (SAM)-functionalised substrates with single molecule contact printing. Nanoscale.

[94] Tian C., Kim H., Sun W., Kim Y., Yin P., Liu H. (2017). DNA nanostructures-mediated molecular imprinting lithography. ACS Nano.

[95] Surwade S.P., Zhou F., Li Z., Powell A., O'Donnell C., Liu H. (2016). Nanoscale patterning of self-assembled monolayers using DNA nanostructure templates. Chem Commun.

[96] Wang Z.-G., Liu Q., Ding B. (2014). Shape-controlled nanofabrication of conducting polymer on planar DNA templates. Chem Mater.

[97] Knudsen J.B., Liu L., A.L. Bank Kodal, Madsen M., Li Q., Song J. (2015). Routing of individual polymers in designed patterns. Nat Nanotechnol.

[98] Surwade S.P., Zhao S., Liu H. (2011). Molecular lithography through DNA-mediated etching and masking of SiO2. J Am Chem Soc.

[99] Surwade S.P., Zhou F., Wei B., Sun W., Powell A., O'Donnell C. (2013). Nanoscale growth and patterning of inorganic oxides using DNA nanostructure templates. J Am Chem Soc.

[100] Jin Z., Sun W., Ke Y., Shih C.-J., Paulus G.L.C., Hua Wang Q. (2013). Metallized DNA nanolithography for encoding and transferring spatial information for graphene patterning. Nat Commun.

[101] Shen B., Linko V., Tapio K., Kostiainen M.A., Toppari J.J. (2015). Custom-shaped metal nanostructures based on DNA origami silhouettes. Nanoscale.

[102] Pillers M.A., Lieberman M. (2014). Thermal stability of DNA origami on mica. J Vac Sci Technol B.

[103] Kim H., Surwade S.P., Powell A., O’Donnell C., Liu H. (2014). Stability of DNA origami nanostructure under diverse chemical environments. Chem Mater.

[104] Gopinath A., Rothemund P.W.K. (2014). Optimized assembly and covalent coupling of single-moleculeDNA origami nanoarrays. ACS Nano.

[105] Kershner R.J., Bozano L.D., Micheel C.M., Hung A.M., Fornof A.R., Cha J.N. (2009). Placement and orientation of individual DNA shapes on lithographically patterned surfaces. Nat Nanotechnol.

[106] Teshome B., Facsko S., Keller A. (2014). Topography-controlled alignment of DNA origami nanotubes on nanopatterned surfaces. Nanoscale.

[107] Linko V., Shen B., Tapio K., Toppari J.J., Kostiainen M.A., Tuukkanen S. (2015). One-steplarge-scale deposition of salt-freeDNA origami nanostructures. Sci Rep.

[108] Kim H., Arbutina K., Xu A., Liu H. (2017). Increasing the stability of DNA nanostructure templates by atomic layer deposition of Al2O3 and its application in imprinting lithography. Beilstein J Nanotechnol.

[109] Matković A., Vasić B., Pešić J., Prinz J., Bald I., Milosavljević A.R. (2016). Enhanced structural stability of DNA origami nanostructures by graphene encapsulation. New J Phys.

[110] Stopar A., Coral L., Di Giacomo S., Adedeji A.F., Castronovo M. (2018). Binary control of enzymatic cleavage of DNA origami by structural antideterminants. Nucleic Acids Res.

[111] Keum J.-W., Bermudez H. (2009). Enhanced resistance of DNA nanostructures to enzymatic digestion. Chem. Commun..

